# Conserved Genoarchitecture of the Basal Hypothalamus in Zebrafish Embryos

**DOI:** 10.3389/fnana.2020.00003

**Published:** 2020-02-06

**Authors:** Theresa Schredelseker, Wolfgang Driever

**Affiliations:** ^1^Developmental Biology, Institute Biology I, Faculty of Biology, University of Freiburg, Freiburg, Germany; ^2^CIBSS and BIOSS – Centres for Biological Signalling Studies, University of Freiburg, Freiburg, Germany

**Keywords:** prosomeric model, bsx brain-specific homeobox, zebrafish brain development, genoarchitecture, patterning, progenitor domains, tuberal hypothalamus, mamillary region

## Abstract

Analyses of genoarchitecture recently stimulated substantial revisions of anatomical models for the developing hypothalamus in mammalian and other vertebrate systems. The prosomeric model proposes the hypothalamus to be derived from the secondary prosencephalon, and to consist of alar and basal regions. The basal hypothalamus can further be subdivided into tuberal and mamillary regions, each with distinct subregions. Albeit being a widely used model system for neurodevelopmental studies, no detailed genoarchitectural maps exist for the zebrafish (*Danio rerio*) hypothalamus. Here, we compare expression domains of zebrafish genes, including *arxa*, *shha*, *otpa*, *isl1*, *lhx5*, *nkx2.1*, *nkx2.2a*, *pax6*, and *dlx5a*, the orthologs of which delimit specific subregions within the murine basal hypothalamus. We develop the highly conserved *brain-specific homeobox* (*bsx*) gene as a novel marker for genoarchitectural analysis of hypothalamic regions. Our comparison of gene expression patterns reveals that the genoarchitecture of the basal hypothalamus in zebrafish embryos 48 hours post fertilization is highly similar to mouse embryos at E13.5. We found the tuberal hypothalamus in zebrafish embryos to be relatively large and to comprise previously ill-defined regions around the posterior hypothalamic recess. The mamillary hypothalamus is smaller and concentrates to rather medial areas in proximity to the anterior end of the neural tube floor plate. Within the basal hypothalamus we identified longitudinal and transverse tuberal and mamillary subregions topologically equivalent to those previously described in other vertebrates. However, the hypothalamic diencephalic boundary region and the posterior tuberculum still provide a challenge. We applied the updated prosomeric model to the developing zebrafish hypothalamus to facilitate cross-species comparisons. Accordingly, we applied the mammalian nomenclature of hypothalamic organization to zebrafish and propose it to replace some controversial previous nomenclature.

## Introduction

The hypothalamus comprises a group of important neuroendocrine and neuromodulatory nuclei. Its organization within the forebrain has been investigated extensively (Puelles et al., 2012; Xie and Dorsky, 2017). Gene expression domains proved a valuable tool in comparative neuroanatomy, since the patterns they produce often remain topologically homologous through evolution, despite considerable shifts occurring in some species. Their analysis thus allows to generate *genoarchitectonic* maps which help in understanding both developmental and evolutionary relationships between forebrain regions (Puelles and Ferran, 2012).

The caudal part of the primary prosencephalon (forebrain) will give rise to the diencephalon, which has been suggested to be subdivided into transverse units called prosomers. The rostral part of the prosencephalon is considered to represent a complex protosegment, termed *secondary prosencephalon* (Puelles and Rubenstein, 2003), and will give rise to both the telencephalon and the hypothalamus.

In contrast to columnar models of brain architecture, which consider the whole hypothalamus to derive from the basal plate (reviewed in Bedont et al., 2015), the prosomeric model proposes a subdivision of the hypothalamus into both alar and basal regions. According to the most recently updated prosomeric model, the hypothalamus can be subdivided from caudal to rostral into peduncular, terminal and acroterminal regions (Puelles et al., 2012; Ferran et al., 2015). The acroterminal region was defined as derivative of the rostral-most neural tube, and fate-mapping experiments revealed its dorsal derivatives to correspond to the anterior commissure region and optic chiasm, while the ventral derivatives include neurohypophysis, arcuate nucleus and finally the median mamillary area (discussed in Puelles et al., 2012).

Within the hypothalamus four longitudinal domains can be distinguished from dorsal to ventral: paraventricular and subparaventricular in the alar plate and tuberal and mamillary in the basal plate (Puelles et al., 2012; Puelles and Rubenstein, 2015). Within the basal hypothalamus, the mamillary regions can further be divided into a more ventral area, consisting of the peduncular retromamillary domain (RM) and the terminal mamillary domain (*sensu stricto*, MA) and a more dorsal region, consisting of the peduncular periretromamillary domain (PRM) and the terminal perimamillary domain (PM). Similarly, the tuberal hypothalamus is subdivided into ventral, intermediate and dorsal components, usually abbreviated as TuV, TuI, TuD and RTuV, RTuI, RTuD for terminal and peduncular subdomains, respectively (Puelles and Rubenstein, 2015).

Most of the updated prosomeric model of the hypothalamus was established based on gene expression patterns in mouse embryonic brain development (Morales-Delgado et al., 2011; Díaz et al., 2014; Morales-Delgado et al., 2014; Ferran et al., 2015). However, being conceptualized as a cross-species vertebrate model, it has also been applied with species-specific adaptations to frogs (Dominguez et al., 2013, 2014; González et al., 2017), reptiles (Moreno et al., 2012, 2017; Dominguez et al., 2015), and catsharks (Santos-Duran et al., 2015, 2018; Santos-Durán et al., 2016) recently. Even though zebrafish represent a widely used model organism for developmental neurobiology, to our knowledge no study as of yet attempted to apply the updated model to zebrafish. In contrast, for the zebrafish larval hypothalamus often a heuristic subdivision into rostral, intermediate and caudal hypothalamus is used (Wullimann et al., 1996; Manoli and Driever, 2014; Biran et al., 2015; Mueller and Wullimann, 2016; Muthu et al., 2016). These anatomical terms of location, however, can be misleading as they refer to actual positions in the embryo and not to their presumptive embryological and genoarchitectonic origin in relation to the neural tube. Since the zebrafish neural tube, however, forms a pronounced cephalic flexure, those two coordinate systems are highly incongruent.

Recent anatomical analyses of the hypothalamus focused on the alar regions (Herget et al., 2014; Nagpal et al., 2018). Even more recently, a novel anatomical entity, the optic recess region (ORR) has been proposed to render the concept of an alar hypothalamus obsolete (Affaticati et al., 2015; Yamamoto et al., 2017; Alié et al., 2018). Very little attention has been paid to the teleost basal hypothalamus for which homology with its mammalian counterpart has been considered to be modest (Vernier, 2017). In contrast, here we apply the updated prosomeric model (Puelles et al., 2012; Puelles and Rubenstein, 2015) to the developing zebrafish basal hypothalamus and find extensive homology.

In our work, we follow the parsimony principle, i.e., demarcate regions assuming the highest grade of homology, and aim to describe a genoarchitectural organization of the zebrafish embryonic hypothalamus postulating as few as possible evolutionary changes. We selected genes whose expression domains in model organisms for other vertebrate classes have been shown to represent defined progenitor domains within the embryonic basal hypothalamus, and analyzed expression of those genes in the zebrafish brain 48 hours post fertilization (hpf), a stage at which the cephalic flexure is fully formed and the major regionalization of the brain is essentially completed (Mueller and Wullimann, 2016).

We were able to identify both tuberal and mamillary regions and therein several subdomains which topologically correspond to those of the updated prosomeric model. When we detected significant differences in gene expression domains between optical sections closer or further away from the midline, we mostly labeled those as “midsagittal,” “intermediate sagittal” or “parasagittal” (Supplementary Figure S1), rather than “periventricular” or “mantle,” to account for ventricular recesses and to avoid confusion with neurogenic zones. If not specified in the image panels, intermediate sagittal sections (Supplementary Figure S1) are shown.

Alongside our genoarchitectural analysis of the basal hypothalamus we characterized the expression pattern of the *brain-specific homeobox* (*bsx*) gene in detail. The expression pattern of this homeobox transcription factor has previously been described in mice (Cremona et al., 2004). This work was, however, prior to the prosomeric model being well-established, and comprehensive reviews of the updated prosomeric model do not discuss *Bsx* (Puelles et al., 2012). In this study we demonstrate the hypothalamic regionalization to be highly conserved throughout evolution and propose that expression of the conserved *bsx* transcription factor might function as a valuable marker for several distinct subdomains therein.

## Materials and Methods

### Animal Strains and Maintenance

Wildtype ABTL zebrafish were kept at 28.5°C on a 14 h light/10 h dark cycle and natural mating was allowed in breeding traps. Embryos were kept in 3 g/l Red Sea salt (Red Sea) at 28.5°C and staged according to Kimmel et al. (1995). Starting from 8 hpf, 0.2 mM N-phenylthiourea (Sigma-Aldrich) was used to prevent pigmentation. All experiments were carried out in accordance with the German Animal Welfare Act.

### Cloning of cDNA Fragments for Probe Synthesis

A list of all transcripts which were used as targets for *in situ* hybridization probes is given in Table 1. For all probes which have been described previously, references are given in Supplementary Table S1. Templates for all other probes were PCR amplified from cDNA and subcloned into pCRII-TOPO plasmid (Invitrogen) as has been described before (Schredelseker and Driever, 2018). Primer sequences are shown in Supplementary Table S1.

**TABLE 1:** All genes for which RNA antisense probes have been used for *in situ* hybridization.

**Abbreviation**	**Full Name**	**Probe Seq**	**NCBI RefSeq**
*agrp*	*agouti related neuropeptide*	68…649	XM_009303347.3
*arxa*	*aristaless related homeobox a*	start < 226…1157	NM_131384.1
*bsx*	*brain-specific homeobox*	38…773	NM_214727.1
*dlx5a*	*distal-less homeobox 5a*	13…507	NM_131306.2
*fgf8a*	*fibroblast growth factor 8a*	1…1461	AF034264.1
*foxb1a*	*forkhead box B1a*	685…1920	NM_131285.1
*isl1*	*ISL LIM homeobox 1*	start < 74…2328	XM_021475997.1
*lef1*	*lymphoid enhancer-binding factor 1*	11…450	AF164700.2
*lhx5*	*LIM homeobox 5*	start < 1185…2016	NM_131218.1
*lhx6*	*LIM homeobox 6*	918…1292	NM_001004015.2
*lhx9*	*LIM homeobox 9*	2466…2698	NM_001037243.2
*nkx2.1*	*NK2 homeobox 1*	156…765	NM_131776.1
*nkx2.2a*	*NK2 homeobox 2a*	33…498	NM_131422.3
*nr5a2*	*nuclear receptor subfamily 5, group A, member 2*	1582…3110	NM_131463.1
*otpa*	*orthopedia homeobox a*	540…1020	NM_001128703.1
*pax6a*	*paired box 6a*	700…2161	XM_009297889.3
*pax7a*	*paired box 7a*	332…2225	AF014368.1
*penka*	*proenkephalin a*	223…861	NM_200083.2
*shha*	*sonic hedgehog signaling molecule a*	79…1757	NM_131063.3
*th*	*tyrosine hydroxylase*	268…1092	NM_131149.1

*Third column indicates the sequence range used for probe generation. Numbering refers to the reference sequence indicated in fourth column. References for published probes and primer sequences for probes which were cloned in this study can be found in Supplementary Table S1.*

### Whole-Mount *in situ* Hybridization

At 48, 72 or 96 hpf manually dechorionated embryos were fixed in 4% PFA/PBST (137 mM NaCl, 2.7 mM KCl, 10 mM Na_2_HPO_4_, 1.8 mM KH_2_PO_4_, 0.1% Tween20) overnight at 4°C before being dehydrated stepwise (25% MeOH/PBST, 50% MeOH/PBST, 75% MeOH/PBST, 5 min each) and stored in MeOH at −20°C before being used for whole-mount *in situ* hybridization. Double-fluorescent whole-mount *in situ* hybridization was carried out as previously described (Filippi et al., 2007; Ronneberger et al., 2012). In very few reactions we observed TSA cross-reactions leading to weak fluorescent labeling of individual probes in not only one but both channels (*bsx* in Figure 5F, *nkx2.1* in Supplementary Figure S5C′ and *bsx* in Supplementary Figure S5D). However, cross-reaction is so weak that it does not obscure the results of the expression analysis.

### Imaging and Figure Preparation

All recordings were made on a Zeiss LSM 880 using an LD-LCI Plan Apochromat 25x/0.8 objective. The Zeiss ZEN Black software was used to make linear adjustments of levels through histogram clipping and to generate maximum intensity projections of selected focal planes. Figures were assembled using Adobe Photoshop CS5 or CS6. Full z-stacks are available upon request.

## Results

### Subdivision of the Basal Hypothalamus in Zebrafish Larvae Into Mamillary and Tuberal Regions

**FIGURE 1:**
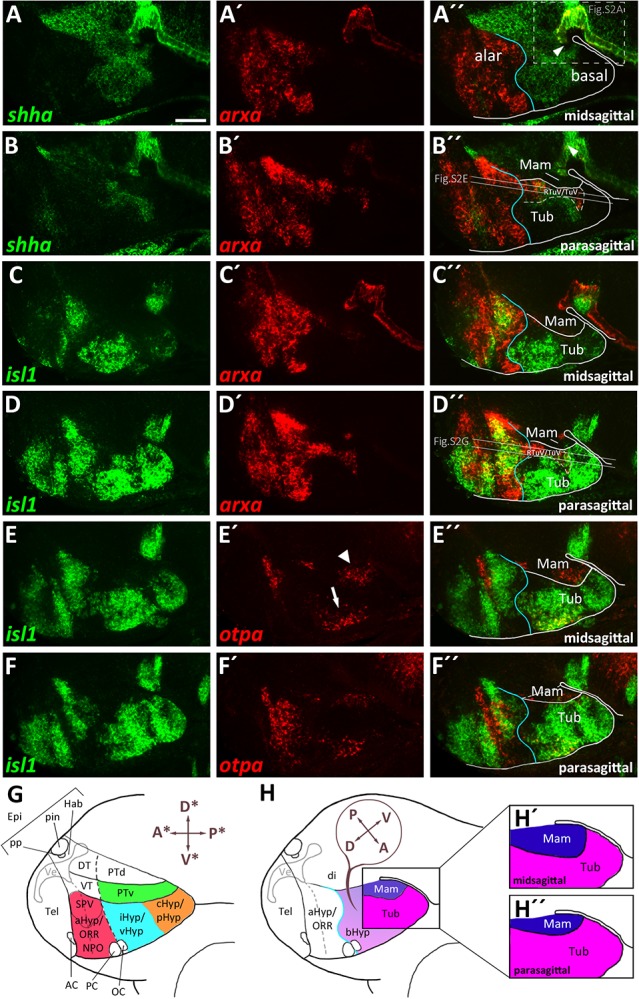
Gene expression domains demarcate alar and basal territories within the secondary prosencephalon as well as tuberal and mamillary subregions within the basal hypothalamus. **(A–F″)** Sagittal optical sections of zebrafish embryos 48 hpf at medial, ventricular or more lateral, mantle positions as indicated, stained by double-fluorescent whole-mount *in situ* hybridization using probes as indicated. **(G)** Integration of several prevalent anatomical models of the zebrafish embryonic forebrain referencing orientation colinear to the body axis (Wullimann et al., 1996; Manoli and Driever, 2014; Biran et al., 2015; Mueller and Wullimann, 2016; Muthu et al., 2016). **(H)** Proposed model of hypothalamus subdivision into mamillary and tuberal areas at ventricular **(H′)** or mantle zones **(H″)** with anatomic directions colinear to the neuraxis. Dashed box in **(A″)** indicates area which is shown in higher magnification in Supplementary Figure S2A. Arrowhead in **(A″)** indicates the rostral border of the floor plate. The alar-basal boundary is shown in cyan. Maximum intensity projections of 10 **(A–A″)**, 15 **(C–C″)**, 25 **(B–B″,D–D″,F–F″),** or 35 **(E–E″)** 1 μm confocal planes. Abbreviations see list. Scale bar 50 μm.

To better understand the cephalic flexure and anterior-posterior organization of the forebrain in zebrafish, we aimed to identify the anterior end of the floor plate in 48 hpf embryos. Given that the floor plate in mouse embryos has been shown to express *Shh* and *Arx* (Cho et al., 2014), we performed double fluorescent *in situ* hybridization using *arxa* and *shha* probes (Figures 1A–B″). Based on comparative neuroanatomical analyses and fate mapping experiments carried out by others (discussed in Puelles et al., 2012), we hypothesized the region around the rostral most end of the *shha*^+^ *arxa*^+^ floor plate (Figures 1A″,B″ and Supplementary Figures S2A–A″, arrowheads) to develop into mamillary regions of the hypothalamus. Beyond the floor plate, *arxa* and *shha* in the secondary prosencephalon are expressed mostly in a mutually exclusive pattern, marking the alar-basal boundary (Puelles et al., 2012; Figures 1A–A″). While *shha* expression in the basal hypothalamus is mainly restricted to medial (i.e., midsagittal or periventricular) regions, *arxa* expression forms a more laterally located band within the basal hypothalamus (Figures 1B–B″ and Supplementary Figures S2E–E″). Based on *Arx* expression in mouse embryonic development (Puelles et al., 2012), we hypothesized this band to correspond to the ventralmost retrotuberal and tuberal areas (TuV/RTuV, Figures 1B–B″,D–D″). We supported this interpretation through the observation that this *arxa*^+^ domain also expresses *lhx6*, defining the TuV/RTuV region (Puelles et al., 2012; Supplementary Figures S2B–B″). This *arxa*^+^, *lhx6*^+^ band is called tuberomamillary terminal by proponents of the columnar model (Shimogori et al., 2010; Supplementary Figure S2H) and in the updated prosomeric model represents the border between the tuberal and mamillary regions (Puelles et al., 2012; Supplementary Figure S2I). *shha* and *arxa* expression reveal that parts of an area surrounding the rostral end of the neural tube, which often has been referred to as “ventral posterior tuberculum region” (“PTv”; Mueller and Wullimann, 2016), actually correspond to mamillary hypothalamus (Figure 1H and Supplementary Figure S2J). Consequently, we considered a quite large area on the other side of the *arxa*^+^, *lhx6*^+^ band to be located embryologically dorsal to mamillary regions, and thus representing tuberal hypothalamus (Figure 1H and Supplementary Figure S2J). This area has previously been labeled “caudal” or “posterior” hypothalamus (Wolf and Ryu, 2013; Xie and Dorsky, 2017; Figure 1G). We confirmed the tuberal nature of this area through the expression of *isl1*, which is moderate in medial regions (Figures 1C–C″), but more pronounced and expanded toward the end of the floor plate in more lateral (i.e., parasagittal, mantle) areas (Figures 1D–D″). We concluded that the *isl1* negative mamillary regions are larger medially and thinner laterally (Figures 1H′–H″). Similar observations have been reported previously for mouse (Puelles et al., 2012). We found *otpa* in the zebrafish basal hypothalamus to be expressed in both tuberal (Figures 1E–E″, arrow in Figure 1E′ and Supplementary Figures S2C–D″) and mamillary regions (Figures 1E–E″, arrowhead in Figure 1E′ and Supplementary Figures S2C–D″). This is in line with murine *Otp* being expressed within the PM/PRM band as well as in dorsal tuberal regions (TuD; Morales-Delgado et al., 2014; Ferran et al., 2015). Again, we observed the mamillary *otpa* expression domain to be larger midsagittally and the *isl1* expressing tuberal regions to surround this domain at parasagittal levels (Figures 1E–F″ and Supplementary Figures S2F–G″). We will discuss the organization of the basal hypothalamus along the radial axis in more details later. With respect to the rostro-caudal and dorso-ventral axis, we will use the terms anterior, posterior, dorsal and ventral related to the neuraxis, unless indicated otherwise (Figure 1H). We propose a subdivision of the embryonic zebrafish basal hypothalamus into tuberal and mamillary regions (Figure 1H) based on conserved genoarchitecture to replace previous models of hypothalamus organization into “anterior” or “rostral,” “intermediate” or “ventral” and “posterior” or “caudal” zones (Figure 1G).

### *bsx* Is Expressed in Both Tuberal and Mamillary Regions of the Basal Hypothalamus

**FIGURE 2:**
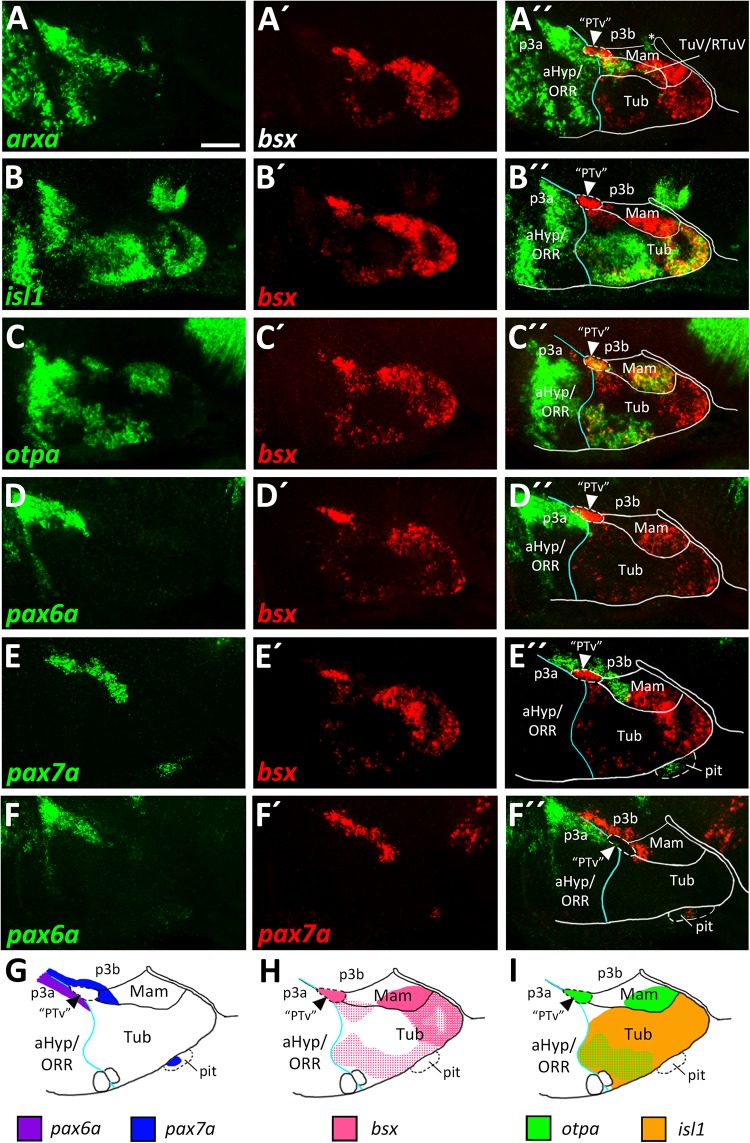
*bsx* expression in relation to other patterning factors within the basal hypothalamus. **(A–F″)** Sagittal optical sections of zebrafish embryos 48 hpf, stained by double-fluorescent whole-mount *in situ* hybridization using probes as indicated. Schematics show expression patterns of *pax6a*, *pax7a* **(G)**, *bsx* **(H)**, *otpa* and *isl1* **(I)** in and close to the basal hypothalamus. Asterisk in **(A″)** indicates *arxa* expression in the floor plate. Arrowheads indicate the domain we operationally define as “PTv.” The alar-basal boundary is shown in cyan. Maximum intensity projections of 20 **(A–B″,D–E″)**, 25 **(F–F″),** or 30 **(C–C″)** 1 μm confocal planes. Abbreviations see list. Scale bar 50 μm.

The *bsx* transcription factor is expressed in several domains within the secondary prosencephalon in mouse and zebrafish (Cremona et al., 2004; Schredelseker and Driever, 2018). Here, comparing *bsx* expression with *arxa* as a marker for the alar territories, we found that most *bsx* expression in the ventral forebrain is restricted to the basal plate (Figures 2A–A″,H). However, a small *bsx* expression domain, located right at the alar-basal boundary, appears to be largely surrounded by *arxa* expression, albeit being *arxa* negative (Figure 2A″, arrowhead). We observed a similar situation when we compared *bsx* expression in this area to *shha* expression, marking basal regions (Puelles et al., 2012): *bsx* expressing cells appeared to be located directly adjacent but not within the *shha* expression area (Figures 5B–B″). *shha* expression is restricted to periventricular zones, while *bsx* is expressed mainly in mantle layers. Therefore, it was difficult to determine if this *bsx* domain belongs to the alar or basal plate. Next, we aimed to resolve if this domain is located within the hypothalamus or within the diencephalon. As a marker for the basal part of the hypothalamus abutting prosomer 3 (p3b) we used *pax7a* (Moreno et al., 2014), the expression of which, however, has been described to reach into the hypothalamus during some stages of amphibian development (Bandín Carazo et al., 2013; Joven et al., 2013). We detected adjacent expression of *pax7a* and *bsx* (Figures 2E–E″). Co-staining of *bsx* and *pax6a*, the latter having been described to be expressed in the alar part of prosomer 3 (p3a; Martinez-Ferre and Martinez, 2012; Moreno et al., 2014), also revealed adjacent expression domains (Figures 2D–D″) rather than overlapping ones. Co-staining *pax6a* and *pax7a* confirmed existence of a domain located right at both, the border between alar and basal plate, and the border between prosomer 3 and hypothalamus (Figures 2F–G′, arrowheads in Figures 2F″,G).

We found that directly lateral to the *bsx* expressing domain in this area, *th* expressing dopaminergic neurons are located (Supplementary Figures S3D–E″). Those *th* expressing cells, previously referred to as the diencephalic cluster 2 (DC2), have been characterized to be located within the “PTv” (Vernier and Wullimann, 2009) and based on its relative position therein have recently been referred to as “anterior rostral posterior tuberculum” dopaminergic cells (“PTar”; Reinig et al., 2017). We thus operationally associated the *bsx* expression in this region with the “PTv.”

Expression of *bsx* on both sides of the *arxa*^+^/*lhx6*^+^ RTuV/TuV band (Figures 2A–A″ and Supplementary Figures S3A–A″) indicated that *bsx* is expressed in both mamillary and tuberal regions. We confirmed *bsx* expression in tuberal regions through a co-staining with *isl1*, which, in line with data from mice (Puelles et al., 2012), we found to be expressed broadly within the tuberal region, therein partly colocalizing with *bsx* (Figures 2B–B″,H,I). In line with our previous results (Figures 1C–F″), we found the *isl1* expression domain to be expanded in parasagittal sections partially overgrowing the mamillary region laterally (Supplementary Figures S3B–C″). In the area containing “PTar” dopaminergic neurons, as well as in mamillary regions we found *bsx* expression to overlap with *otpa* expression (Figures 2C–C″,H,I). In the tuberal hypothalamus we also found *bsx* to colocalize with *otpa*, but additionally to be expressed in *otpa* negative tuberal areas (Figures 2C–C″,H,I). We will discuss these and other tuberal subregions in more detail in another section. We conclude that similarly to what has been described in mouse (Cremona et al., 2004), *bsx* is expressed in both tuberal and mamillary regions within the basal hypothalamus suggesting that *bsx* expression is highly conserved in the secondary prosencephalon.

### The Mamillary Region Can Be Subdivided Into Four Territories

**FIGURE 3:**
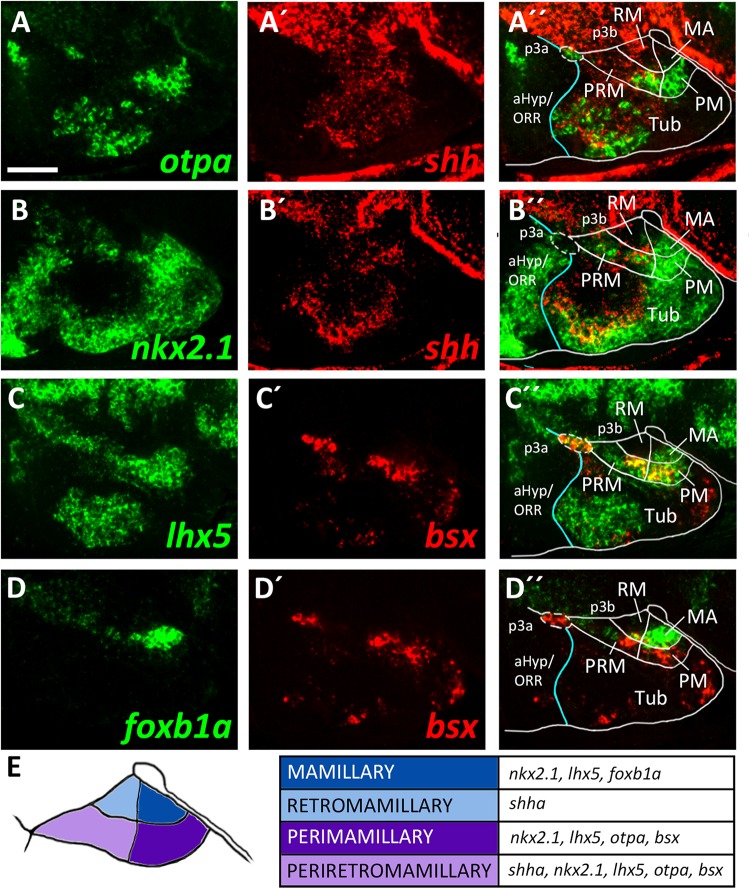
Longitudinal and transverse subdivisions of the mamillary regions based on conserved gene expression domains. **(A–D″)** Sagittal optical sections of ventricular hypothalamic regions in zebrafish embryos 48 hpf, stained by double-fluorescent whole-mount *in situ* hybridization using probes as indicated. **(E)** Schematic showing subdomains of the mamillary region and the genes expressed therein. The alar-basal boundary is shown in cyan. Maximum intensity projections of 10 **(C–D″)**, 15 **(A–A″)**, or 30 **(B–B″)** 1 μm confocal planes. Abbreviations see list. Scale bar 50 μm.

To identify mamillary subregions based on gene expression patterns described in other model organisms, we analyzed the expression domains of *shha*, *nkx2.1*, *otpa*, *foxb1a*, *lhx5* and *bsx*. To confirm that *otpa* is expressed in both the peduncular PRM and terminal PM, we analyzed its expression relative to *shha*, which in mouse labels the peduncular PRM and RM domains but not the terminal mamillary regions, PM and MA (Puelles et al., 2012). In line, we found *otpa* and *shha* expression to overlap in a posterior, dorsal mamillary domain which we assume to correspond to PRM (Figures 3A–A″). We found that an area closer toward the floor plate, thus embryologically ventral, expresses *shha* but not *otpa* (Figures 3A–A″) and reasoned that this domain should be considered RM. Consistent with data from mouse (Puelles et al., 2012), the mamillary *otpa* expression domain extends anteriorly (in relation to the neuraxis) into a *shha* negative area, presumably representing PM (Figures 3A–A″).

To confirm this model, we compared expression of *shha* to the expression of *nkx2.1* which in mice is expressed in all mamillary regions except RM (Puelles et al., 2012). This allowed us to reinforce RM as a distinct domain expressing *shha* but not *nkx2.1* and PRM as expressing both *shha* and *nkx2.1* (Figures 3B–B″). We further concluded that the *nkx2.1*^+^, *shha*^–^ areas encompass the terminal mamillary domains PM and MA (Figures 3B–B″). As we previously showed *bsx* expression to perfectly overlap with *otpa* expression in the mamillary regions (Figures 2C–C″), we used *bsx* and *otpa* interchangeably as markers for the PRM/PM band. *lhx5* has recently been described in catshark as being expressed in PRM, PM and MA but not in RM (Santos-Duran et al., 2018). Co-staining of *lhx5* and *bsx* suggested that this expression pattern is conserved in zebrafish and that *bsx* is expressed in PRM as well as PM but not in MA (Figures 3C–C″). To support this hypothesis, we used *foxb1a* as a marker for the MA region (Zhao et al., 2008; Puelles et al., 2012) and found expression close to the end of the floor plate in an area which does not express *bsx* (Figures 3D–D″). We concluded that in the embryonic zebrafish hypothalamus, mamillary regions can be defined and divided into four subdomains corresponding to those previously described in mammals (Figure 3E; Puelles et al., 2012).

### The Mamillary Domain Is Larger in Medial Regions and Flanked by Both Perimamillary and Tuberal Domains in Lateral Regions

**FIGURE 4:**
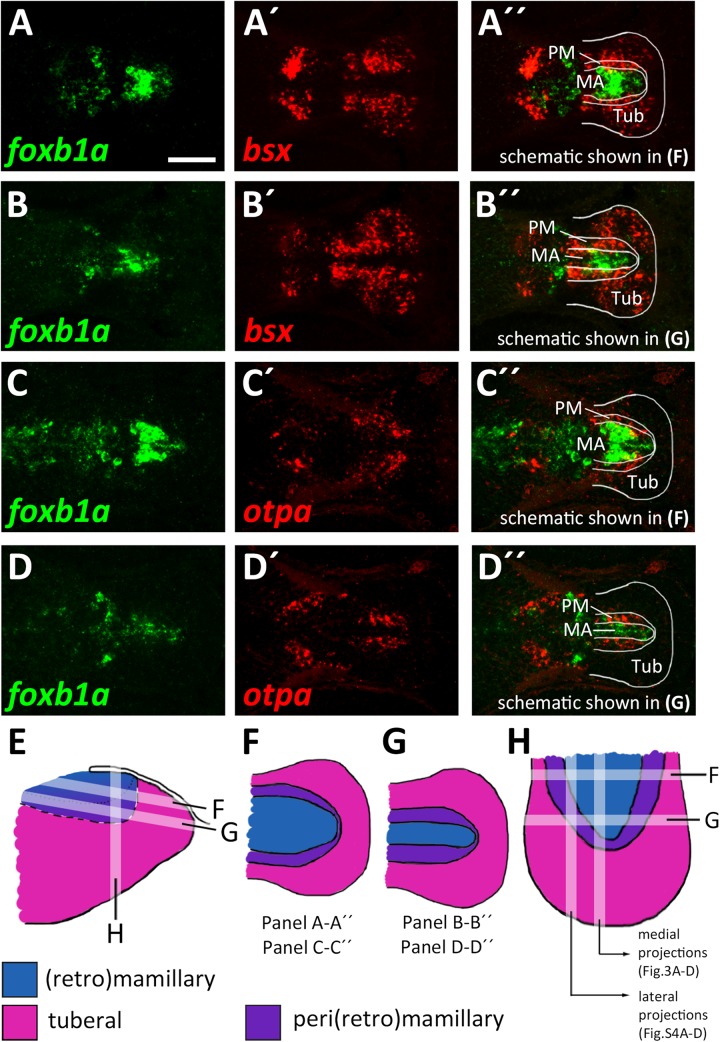
Radial organization of the basal hypothalamus. **(A–D″)** Horizontal optical sections of ventricular hypothalamic regions in zebrafish embryos 48 hpf, stained by double-fluorescent whole-mount *in situ* hybridization using probes as indicated. **(E)** Schematics showing lateral view on basal hypothalamus with tuberal and mamillary regions color coded as indicated. Section planes of schematics representing horizontal **(F,G)** and sagittal **(H)** projections are shown. Maximum intensity projections of 10 **(A–B″,D–D″)** or 15 **(C–C″)** 1 μm confocal planes. Abbreviations see list. Scale bar 50 μm.

The model which we unfolded in the previous section shows the spatial organization of the mamillary subregions within medial regions of the basal hypothalamus, i.e., those regions that lie close to the ventricle. We noted, however, that the borders shown therein are shifted as we analyzed lateral regions in parasagittal optical section. In those regions further away from the midline which do not express *shha* (Supplementary Figures S4A–B″), we found expression of markers indicative for the PRM/PM band, such as *otpa* (Supplementary Figures S4A,A″), *lhx5* and *bsx* (Supplementary Figures S4C–C″) to reach closer to the floorplate, i.e., embryologically more ventral. On the other hand, the *foxb1a* expression domain, indicative for the MA, was considerably smaller in lateral areas (compare Figures 3D-D″ and Supplementary Figures S4D–D″). Dorsal views confirmed the *foxb1a* expression domain to reach more laterally in ventral regions (Figures 4A–A″,C–C″,F, planes indicated in Figures 4E,H) and to be restricted to medial regions more dorsally (Figures 4B–B″,D–D″,G, planes indicated in Figures 4E,H). Using *otpa* as a marker, we found the PM to surround the *foxb1a* expressing MA area in a cup-like shape (Figures 4C–D″,H). Consistent with this model and with our previous observations, we found *bsx*, which is expressed in both PM and tuberal regions, to be expressed as a broader band surrounding the MA (Figures 4A–B″). We summarized our model of the radial organization within this hypothalamic area in Figures 4E–H.

### The Zebrafish Larval Tuberal Hypothalamus Can Be Subdivided Into Longitudinal and Transverse Subterritories

**FIGURE 5:**
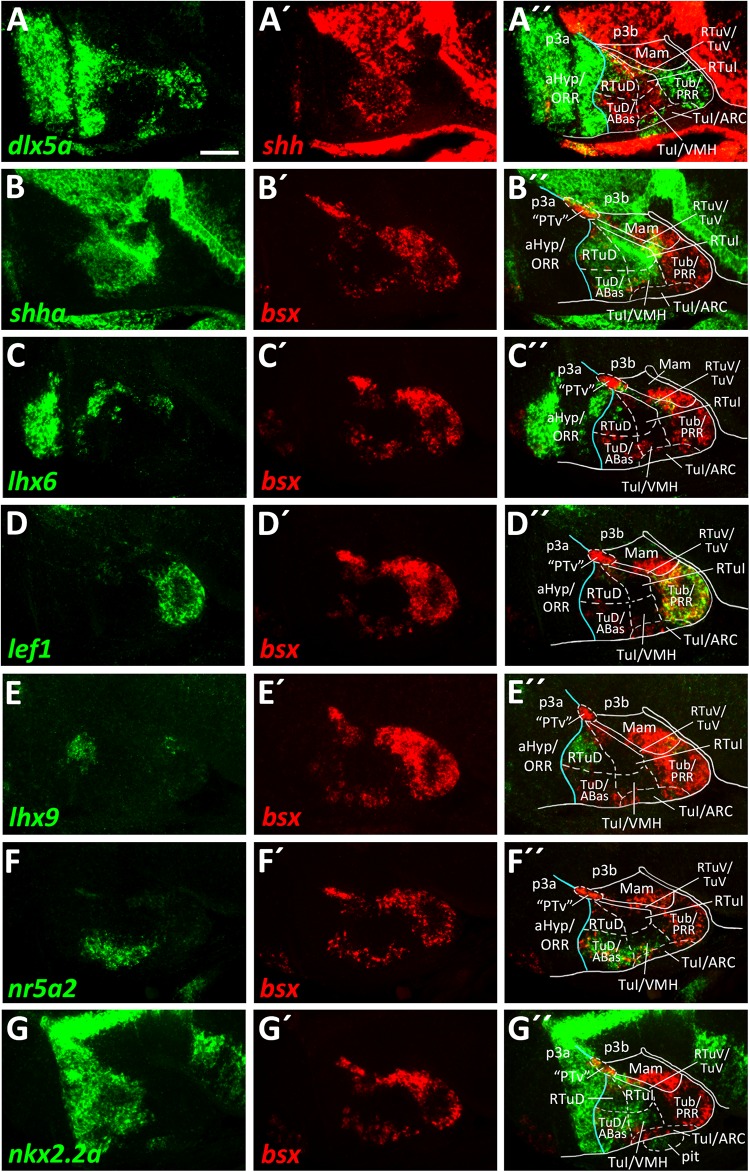
Longitudinal and transverse subdivisions of the tuberal hypothalamus based on conserved gene expression domains. **(A–G″)** Sagittal optical sections of ventricular hypothalamic regions in zebrafish embryos 48 hpf, stained by double-fluorescent whole-mount *in situ* hybridization using probes as indicated. The alar-basal boundary is shown in cyan. Maximum intensity projections of 20 **(E–E″)**, 30 **(A–A″,C–C″,G–G″)**, 40 **(D–D″,F–F″),** or 50 **(B–B″)** 1 μm confocal planes. Abbreviations see list. Scale bar 50 μm.

We already showed that in zebrafish an *arxa*^+^, *lhx6*^+^ band extends from alar regions into the basal hypothalamus (Figures 2A–A″, Supplementary Figures S2B–J, S3A–A″). Following our aim to find the most parsimonious model in terms of homologous gene expression domains marking conserved progenitor domains in the basal hypothalamus, we considered this domain to correspond to RTuV/TuV (Figures 5C–C″). We examined tuberal *shha* expression, which has been shown to extend into terminal regions in TuD (Puelles et al., 2012) and in shell regions of the ventromedial hypothalamus area (VMH; Morales-Delgado et al., 2011).

**FIGURE 6:**
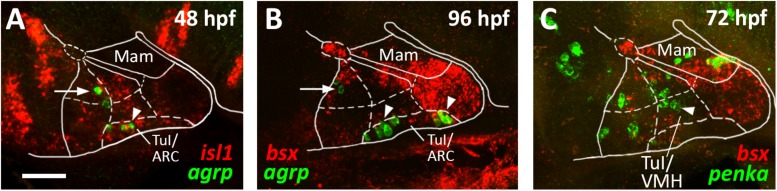
*agrp* and *penka* expression in tuberal subregions. Sagittal optical sections of hypothalamic regions in zebrafish embryos 48 hpf **(A)**, 96 hpf **(B),** or 72 hpf, stained by double-fluorescent whole-mount *in situ* hybridization using probes as indicated. White arrowheads in **(A,B)** indicate *agrp*^+^ cells in the putative arcuate nucleus region; white arrows indicate *agrp*^+^ neurons in retrotuberal territories. White arrowhead in **(C)** indicates *penka*^+^ cells in the putative ventromedial hypothalamus region. The alar-basal boundary is shown in cyan. Maximum intensity projections of 20 **(A)**, 30 **(B)**, or 50 **(C)** 1 μm confocal planes. Abbreviations see list. Scale bar 50 μm.

In line with this, we observed a broad band of *shha* expression which reaches the acroterminal domain close to the alar-basal boundary (Figures 5A′,B). In mouse, where this *Shh* expression domain is conserved, this area is named TuD or anterobasal region (ABas). Topologically ventral to this, *shha* expression was also observed in terminal regions but did not reach the acroterminal end of the forebrain. This domain, just like the TuD/ABas domain, also expresses *nr5a2* (Figures 5F–F″). Since the hypothalamic expression domain of *nr5a2* has been demonstrated to overlap with *nr5a1a* expression (Kurrasch et al., 2007), the mouse homolog of which has been described to be selectively expressed in the TuD/ABas and VMH regions around E13.5 (Puelles et al., 2012; Ferran et al., 2015), we operationally defined this region as VMH. The presence of *penka* expression (Figure 6C) supports our interpretation that this region shares homology with the mammalian VMH. In line with the literature (Morales-Delgado et al., 2011), we found that both the terminal TuD/ABas and VMH domain are the only tuberal regions which are *dlx5a* negative (Figure 5A). Furthermore, we found that the *nr5a2*^+^, *dlx5a*^–^ TuD/ABas and VMH regions also expresses *otpa* (Figures 3A–A″). *Otp* expression in this tuberal subregion has previously been described in mammals (Morales-Delgado et al., 2011), amphibians and reptiles (Dominguez et al., 2015). The same area also expresses *lhx5* (Figures 3C–C″), which has previously been reported to be expressed in a homologous region in catshark (Santos-Duran et al., 2018).

In a large tuberal area embryologically ventral to this (the previously named “cHyp” area as indicated in Figure 1G), we found no *shha* expression (Figures 5A–B″). Absence of *shha* expression as well as presence of *lef1* expression have previously been described for the remaining parts of the terminal intermediate tuberal (TuI) domain (excluding the VMH area; Ferran et al., 2015). Based on *lef1* expression in this area (Figures 5D–D″ and Supplementary Figures S5E–E″), we assume that this area shares some homology with mammalian TuI parts. However, we would like to note that this region in teleosts has features which are absent from the mammalian TuI region, most notably the posterior hypothalamic recess (PR; Mueller and Wullimann, 2016; Xavier et al., 2017; Yamamoto et al., 2017). We hypothesize that the PR might cause the displacement of the *lhx6*^+^ TuV domain to far parasagittal regions (Supplementary Figure S6). Notably, the posterior recess region (PRR) also shows features characteristic for the TuV domain, i.e., the presence of histaminergic neurons at embryonic stages (Eriksson et al., 1998). To highlight our observation that this regions has tuberal (both TuI and TuV) characteristics, we designated this area Tub/PRR.

We also detected a thin band of *fgf8a* expression reaching from an embryologically anterior part of the PM area to an area just above the pituitary (Supplementary Figures S2B–J) and concluded that it represents parts of the acroterminal domain as has been suggested previously in mice (Ferran et al., 2015).

We found *lhx9* expression in a peduncular dorsal tuberal domain close to the alar-basal boundary (Figures 5E–E″), similarly to what has previously been reported for mouse (Puelles et al., 2012). This region, in contrast to the terminal TuD parts, expresses *dlx5a* (Figures 5A–A″ and Supplementary Figures S5A–B″,F,F′) and not *nr5a2* (Figures 5F–F″). Following the updated prosomeric model we thus distinguished between a peduncular *lhx9*^+^, *dlx5a*^+^, *nr5a2*^–^ RTuD domain and a terminal *lhx9*^–^, *dlx5a*^–^, *nr5a2*^+^ TuD domain.

We observed that the zebrafish RTuD and RTuI domains do not express *nkx2.1* (Figures 3B–B″ and Supplementary Figures S5C–C″), while in mouse *nkx2.1* expression was found in all tuberal subregions (Puelles et al., 2012). However, previous work from our group showed that both *nkx2.4a* and *nkx2.4b* are expressed in this region and that those genes act partially redundantly (Manoli and Driever, 2014), suggesting that *nkx2.4* gene products substitute *nkx2.1* gene product functions therein.

We found *nkx2.2a* expression along the alar-basal boundary but also in both RTuD and TuD (Figures 5G–G″). *Nkx2.2a* expression in mice has been shown to be restricted to the alar-basal boundary regions in early stages but starting around E13.5 to expand into more ventral tuberal regions, contributing to both the developing VMH and peduncular dorsomedial hypothalamic nucleus (DMH; Puelles et al., 2012), a derivative of the RTuI domain. This reflects previous descriptions of *nkx2.2a* expression in the zebrafish VMH (Kurrasch et al., 2007) and helped us to identify a border between the peduncular and terminal region within the intermediate tuberal domains. We concluded that *nkx2.2a* is expressed in RTuD and RTuI as well as in the terminal TuD/ABas region and VMH region. The remaining (non-VMH) terminal Tub/PRR region, which expresses *lef1* (Figures 5D–D″; Ferran et al., 2015), as well as an acroterminal domain beneath the VMH area, both do not express *nkx2.2a* (Figures 5G–G″).

We noted that the acroterminal *nkx2.2a*^–^ region is the same area which appears to be mainly *shha* negative. Based on this gene expression profile and on the location of this region right above the pituitary, we hypothesized that it might correspond to the developing arcuate nucleus (ARC). The ARC is anatomically often defined through expression of its hallmark neuropeptides. While it has been shown that *Pomc* expressing neurons actually originate in the TuD/ABas region and only secondarily migrate into the ARC region (Díaz et al., 2014), *Agrp* expressing neurons have been described as to originate directly in the ARC (Díaz et al., 2014). We thus assessed *agrp* expression within the *isl1* expressing tuberal hypothalamus at 48 hpf (Figure 6A, arrowhead) and found several *agrp*^+^ cells within this acroterminal region adjacent to the pituitary. At 96 hpf we found *agrp* expressing neurons spread over a longer part of the acroterminal domain (Figure 6B, arrowhead) and hypothesized this region to represent a structure homologous to the mammalian ARC, similarly to what has previously been suggested (Forlano and Cone, 2007). We also observed few *agrp* expressing cells within the RTuD region (Figures 6A,B, arrow), which, to our knowledge, were never observed in the mammalian hypothalamus and which might express *agrp* only transiently as they were not described in later larval stages (Shainer et al., 2017).

**FIGURE 7:**
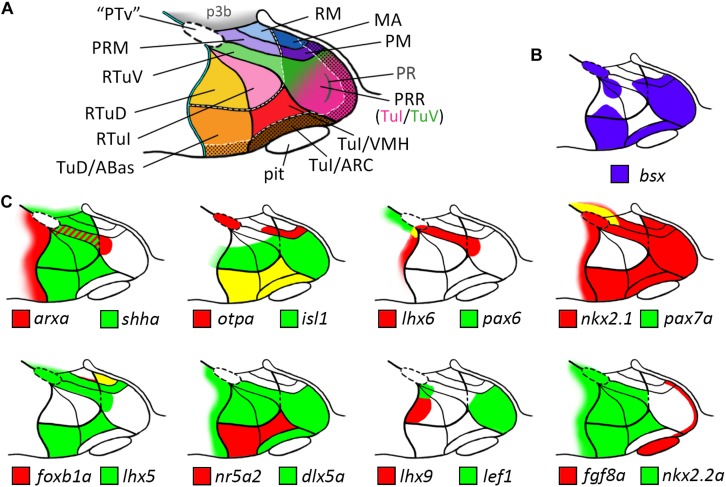
Schematic representations of gene expression patterns in subdomains of the basal hypothalamus. **(A)** Schematic of a representative intermediate sagittal optical section highlighting all subdomains of the basal hypothalamus identified in this study. The alar-basal boundary is shown as thin cyan line with black border. White hatched lines indicate transverse boundaries between the peduncular and terminal hypothalamus as well as between the terminal (solid colors) and acroterminal (shown with black dotted overlay pattern) domains. The green/pink hatched gradient fill area indicates the mixed TuV- and TuI-like characteristics of the PRR (see main text for details). **(B,C)** Expression patterns of **(B)** *bsx* in purple and **(C)** other genes in red and green as indicated within the subdomains of the basal hypothalamus as depicted in **(A)**, yellow indicates overlapping expression. The red-green hatched area in **(C)** indicates expression at different sagittal levels, thus no colocalization. Abbreviations see list.

Our analysis allowed us to establish a map for the embryonic zebrafish basal hypothalamus, showing the subdivisions as defined within the prosomeric model (Figure 7A) and to describe the expression pattern of the *bsx* gene within these regions (Figure 7B). We found *bsx* to be expressed in the tuberal Tub/PRR, ARC and TuD/ABas regions while the VMH area as well as the retrotuberal areas are mainly *bsx* negative with the exception of some dispersed cells. In the perimamillary regions as well as in the posterior tubercular area containing dopaminergic “PTar” neurons, we found *bsx* expression to perfectly overlap with *otpa* expression. We summarized the expression of all other analyzed genes within the basal hypothalamus in Figure 7C.

## Discussion

The extensive utilization of gene expression data has revolutionized our understanding of the evolutionary conserved genoarchitecture of the hypothalamus in mammals and other vertebrate classes, resulting in an updated prosomeric model of hypothalamic organization (Puelles et al., 2012; Puelles and Rubenstein, 2015). However, so far this highly successful approach has not been fully extended to teleosts, and specifically the zebrafish model, which is widely used by neurobiologists to study development and function of the hypothalamus (Löhr and Hammerschmidt, 2011; Xie and Dorsky, 2017). The updated prosomeric model of hypothalamic organization has largely been built upon data from the Allen Developing Mouse Brain Atlas^1^, containing E11.5, E13.5, and E15.5 expression patterns of many genes involved in hypothalamic development. For both the embryonic and adult zebrafish brain several online atlas projects have been initiated by our group and others (Ronneberger et al., 2012; Randlett et al., 2015; Tabor et al., 2019). However, these anatomical gene expression frameworks are largely restricted to late embryonic and early larval stages, when most of the early patterning markers cease their broad expression and may be maintained only in a subset of differentiated neurons, making the assessment of genoarchitecture more difficult. Our experience with the VibeZ expression data registration and 48 hpf anatomical reference framework (Ronneberger et al., 2012) revealed that proper anatomical registration in praxis is very sensitive to variations in cephalic flexure, which tend to generate artifacts during elastic registration especially in the mamillary and tuberal hypothalamus. Therefore, the classical neuroanatomical approach using pairwise mapping of gene expression domains by double fluorescent *in situ* hybridization was preferred in our study.

For analyzing the genoarchitecture of the embryonic basal hypothalamus, we chose 48 hpf since at this stage the major morphogenetic reorganization of the embryonic CNS is accomplished, and local organizing centers have established regional identities in the brain (Mueller and Wullimann, 2016). Importantly, many transcription factor genes involved in pattern formation are still broadly expressed in their anatomical domains 48 hpf. While we did not compare different stages of zebrafish hypothalamus development extensively enough to hypothesize about a potential phylotypic stage, we found marker gene expression domains in most cases to be topologically equivalent in mouse E13.5 and zebrafish 48 hpf brains. In general, our data revealed the genoarchitecture of the embryonic basal hypothalamus to be highly conserved between zebrafish and mouse, i.e., to consist of topologically equivalent subregions defined through homologous gene expression domains (Figure 7). It will be interesting to see how this conservation relates to the anatomical location of neuropeptidergic and neuromodulatory cell types, many of which were recently mapped for the mouse model (Díaz et al., 2014). Proponents of the updated prosomeric model repeatedly pointed out that too little attention has been paid to the cephalic flexure, and to the resulting discrepancies in embryological terms of location (Puelles and Rubenstein, 2003; Puelles et al., 2012). We believe that this is particularly true for zebrafish. To illustrate this, we would like to use the PRR as an example. Neuroanatomical studies, published more than 20 years ago, already suggested this area to correspond to tuberal hypothalamus (Wullimann et al., 1996) but used the term *caudal* Hypothalamus to refer to the location of this area relative to the body axis and not the neuraxis. Synonymously, recent papers often use the term “*posterior* hypothalamus” to refer to this brain structure (Wolf and Ryu, 2013; Muthu et al., 2016). This nomenclature could lead to misconceptions as it might be associated with the posterior nucleus of the hypothalamus which is actually a derivative of the mamillary hypothalamus (Puelles et al., 2012). Based on its relative location to the anterior end of the floorplate, the expression of multiple genuinely tuberal markers, such as *dlx5a* or *isl1*, and the absence of mamillary markers, such as *lhx5*, we classified this region as tuberal.

We found the tuberal part of the PRR to be quite large and to overgrow parts of the mamillary hypothalamus. While also in mice the mamillary regions have been described to be broader in medial regions and thinner in lateral regions (Puelles et al., 2012; Puelles, 2019), the difference in size appears to be even more pronounced in zebrafish. It has been proposed by others to conceptualize the hypothalamus to consist of “modules,” which are gained or lost during evolution (Xie and Dorsky, 2017). Tetrapods have only one hypothalamic recess (of the 3rd ventricle) which is thought to be homologous to the lateral recess in teleosts (Yamamoto et al., 2017). An additional posterior hypothalamic recess, however, is present in several bony (Parent and Northcutt, 1982; Kotrschal et al., 1985) and cartilaginous fish (Meurling and Rodríguez, 1990; Carrera et al., 2012; Rodríguez-Moldes et al., 2017). The PRR in zebrafish is densely populated by CSF-c neurons, many of which contain monoamines such as dopamine and serotonin (Xavier et al., 2017). In zebrafish, histaminergic neurons are located in the PRR (Eriksson et al., 1998) and were associated with a “posterior paraventricular organ” in the adult brain (Kaslin and Panula, 2001). In mammals, the histaminergic neurons are located in the premamillary nucleus (Panula et al., 1984) and were suggested to originate in the RTuV/TuV region (Puelles et al., 2012). Notably, the RTuV/TuV has been associated with a hypothalamic ventricular organ, a “linear circumventricular specialization which is unremarkable in mammals” (Puelles and Rubenstein, 2015). The zebrafish PRR exhibits a high degree of continuous neurogenesis (Grandel et al., 2006; Wang et al., 2012). This might cause the overgrowth of the tuberal PRR and the peculiar shape of the RTuV/TuV domain which could be explained through anisotropic growth (Fu et al., 2019) of tuberal regions as the PRR bulges outward. To account for its divergent phylogenetic status, we suggest for this brain region not to adopt the mammalian nomenclature but to refer to it as PRR.

An alternative or additional explanation for the unexpected shape of the TuV domain as inferred from *lhx6* and *arxa* expression may be considered, which is a fundamental limit of the histogenetic markers used in this and other studies. Genes such as *lhx6* and *arxa* are transiently expressed in multiple developing neurons during progenitor states and maintained in only a small subset of maturing neurons. It is thus possible that cells in the anterior parts of the ventral tuberal regions (Supplementary Figure S2J, gray hatched area labeled “hyp. TuV”) ceased to express *lhx6* and *arxa* or migrated to other regions.

A fundamental anatomical landmark, the alar-basal boundary, in 48 hpf zebrafish larvae has recently been proposed in a way that the *shha* expression within the secondary prosencephalon reaches into the alar plate (Affaticati et al., 2015; Vernier, 2017). We slightly revise this model to make it more parsimonious and propose the alar-basal boundary to coincide with the boundary between the *shha* and *arxa* expression domains, thus proposing the *shha* expression in the secondary prosencephalon to be entirely basal, similar to what has been suggested in mouse (Puelles et al., 2012) and catshark (Santos-Duran et al., 2015).

A more complicated boundary, in our eyes, is the boundary between the hypothalamus and the diencephalon, i.e., prosomer 3. This boundary region has been referred to as posterior tuberculum and has been of special interest because of dopaminergic cell populations, traditionally named diencephalic clusters (DCs), being located within or in proximity to this region (Rink and Wullimann, 2002). By comparing *bsx* expression to the expression of mamillary markers such as *lhx5* and *otpa* and also to the expression of *th*, we were able to resolve the location of some of those dopaminergic cell clusters. *Bsx* and *Otp* expression colocalization has previously been shown in mouse in a structure called “ventral mamillary body” (Cremona et al., 2004). Furthermore, colocalization of *otpa* and *th* expression within the mamillary regions has been demonstrated in Xenopus (Dominguez et al., 2014).

The DC2, 4, 5, and 6 clusters have been shown to be homologous to the A11 dopaminergic system based on their dependency on Otp (Ryu et al., 2007) and their projection pattern (Tay et al., 2011). Ryu et al. (2007) already demonstrated that in mice, A11 neurons are Nkx2.1 positive, despite many of them being localized in thalamic areas, arguing for a potential migration of progenitors from the hypothalamus to the thalamus. Mouse A11 neurons are distributed in the posterior and dorsal hypothalamic areas as well as in thalamic regions (Smeets and González, 2000). Based on our colocalization analysis we consider DC5 and 6 to be located within mamillary *bsx* and *otpa* expressing regions. It is tempting to speculate that DC5 and 6 might be homologous to A11 neurons of the hypothalamus, while DC2 and potentially also DC4 might correspond to A11 neurons which in mammals have been suggested to migrate into thalamic regions, thereby possibly crossing p3b. In catshark, *th* expressing cells in a similar region of the brain have been interpreted as being located on both sides of the boundary between hypothalamic mamillary areas and p3b (Santos-Duran et al., 2015).

Based on the data we present here, we could not determine the exact location of the DC2 and DC4 dopaminergic neurons, which differentiate already at neural plate stages (Mahler et al., 2010). This is well before the complex genoarchitectural pattern which we described for the 48 hpf zebrafish embryo is established. Most genoarchitectural markers are expressed in progenitor cells residing close to the ventricle, and their expression often does not extend into the far lateral mantle regions harboring mature DC2 and DC4 dopaminergic neurons. For the area in which DC2 neurons are located in mantle regions, we found *bsx* expression more medially in intermediate sagittal optical sections. The anatomical status of this area, located both at the alar-basal boundary and at the diencephalic-hypothalamic border, remains challenging. To avoid premature conclusions about this area, we operationally labeled it with “PTv.” However, further studies are needed and might suggest a hypothalamic, prethalamic (p3a) or diencephalic tegmental (p3b) identity of this area. In fact, if the tegmental nature of this area will be refuted in future studies it should no longer be referred to as “posterior tuberculum,” a structure which has been tightly associated with p3b (Mueller and Wullimann, 2016). We hope that the framework we generated in this study will provide a solid ground for future work in determining both origin and destination of DC2 and 4 dopaminergic cells, as well as the exact location of the hypothalamic-diencephalic boundary, which has long been and still is subject of lively discussion among zebrafish neuroanatomists (Biran et al., 2015; Yamamoto et al., 2017).

Genoarchitectonic maps have been worked out for the hypothalamus mainly based on expression data generated on mouse brain slices (Puelles et al., 2012; Díaz et al., 2014; Ferran et al., 2015). These maps provide great tools to facilitate understanding of a complex three-dimensional brain structure. Here, we have extended this model to the evolutionary distant zebrafish embryo, which is an excellent experimental system for the analysis of brain patterning mechanisms, but also for studies of neural circuit formation and function. The gross anatomical differences between fish and mammals have so far hindered the comparison of regulatory circuitries involving the hypothalamus. The emerging shared genoarchitecture of both systems will facilitate the transfer of insights into developmental mechanisms as well as neural circuitries.

## Data Availability Statement

All datasets generated for this study are included in the article/Supplementary Material.

## Ethics Statement

The animal study was reviewed and approved by the Regierungspraesidium Freiburg.

## Author Contributions

TS conceptualized and designed the study and performed the experiments, assembled the figures, and wrote the first draft of the manuscript. WD contributed to design, supervision and editing, and provided project administration and funding acquisition.

## Conflict of Interest

The authors declare that the research was conducted in the absence of any commercial or financial relationships that could be construed as a potential conflict of interest.

## Footnotes

1 http://developingmouse.brain-map.org

## Abbreviations

A: anterior (colinear to neuraxis)
A^∗^: anterior (colinear to body axis)
ABas: anterobasal region
AC: anterior commissure
aHyp: alar hypothalamus
ARC: arcuate nucleus
bsx: brain-specific homeobox
cHyp: “caudal hypothalamus” (1)
D: dorsal (colinear to neuraxis)
D^∗^: dorsal (colinear to body axis)
DC: diencephalic cluster (of dopaminergic neurons) (2)
DM: dorsomedial nucleus region
DT: “dorsal thalamus” (1)
Epi: epithalamus
Hab: habenula
hpf: hours post fertilization
Hyp: hypothalamus
iHyp: “intermediate hypothalamus” (1)
LR: lateral recess
MA: mamillary domain (*sensu stricto*)
Mam: mamillary regions (*sensu lato*)
MM: mamillary region (columnar model) (3)
NPO: neurosecretory preoptic region
OC: optic commissure
OR: optic recess
ORR: optic recess region
P: posterior (colinear to neuraxis)
P^∗^: posterior (colinear to body axis)
PC: posterior commissure
pHyp: “posterior Hypothalamus” (1)
pin: pineal gland
pit: pituitary gland
PM: perimamillary domain
pp: parapineal organ
PreM: premamillary region (3)
PRM: periretromamillary domain
PR: posterior (hypothalamic) recess
PRR: posterior recess region
PTar: “posterior tuberculum anterior rostral” dopaminergic neurons (1)
PTd: “posterior tuberculum dorsal part” (1)
pTh: prethalamus
PTv: “posterior tuberculum ventral part” (1)
p3a: prosomer 3 alar part
p3b: prosomer 3 basal part
RM: retromamillary domain
RTuD: retrotuberal domain dorsal part
RTuI: retrotuberal domain intermediate part
RTuV: retrotuberal domain ventral part
SMM: supramammillary nucleus region (3)
SPV: supraopto-paraventricular region
Tel: telencephalon
TT: tuberomamillary terminal (3)
Tub: tuberal regions (*in sensu lato*)
TuD: tuberal domain dorsal part
TuI: tuberal domain intermediate part
TuV: tuberal domain ventral part
V: ventral (colinear to neuraxis)
V^∗^: ventral (colinear to body axis)
Ve: ventricle
vHyp: “ventral hypothalamus” (1)
VMH: ventromedial hypothalamic nucleus region
VT: “ventral thalamus” (1)
