# Supplementary material for: Conserved Genoarchitecture of the Basal Hypothalamus in Zebrafish Embryos
Source: Front Neuroanat. 2020 Feb 6;14:3. doi: 10.3389/fnana.2020.00003 (PMC7016197; doi:10.3389/fnana.2020.00003)
Supplement: Supplementary file 1 [file Data_Sheet_1.pdf]

Supplementary Material

Conserved Genoarchitecture of the Basal Hypothalamus in Zebrafish Embryos

Frontiers in Neuroanatomy

T. Schredelseker and W. Driever

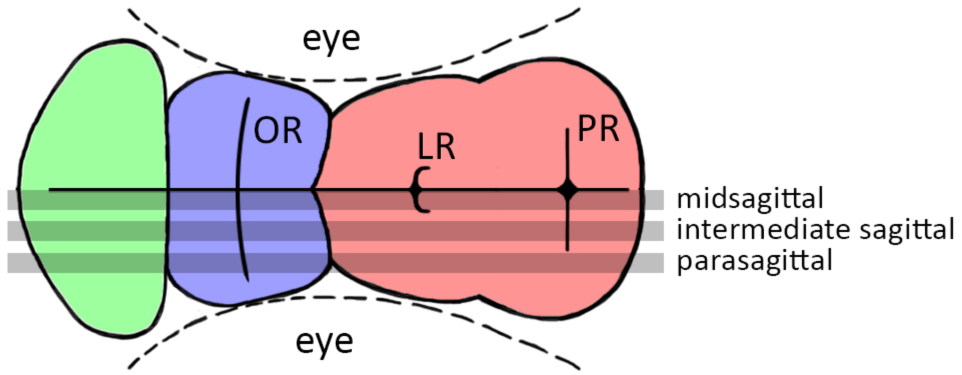

Figure S1: Schematic showing different sagittal section levels and ventricular recesses in the zebrafish brain 48 hpf. green – telencephalon; blue – optic recess region; red – basal hypothalamus; OR – optic recess; LR – lateral recess; PR – posterior recess. Grey shadings indicate approximate optical section levels of which maximal intensity projections are shown in image panels throughout the article.

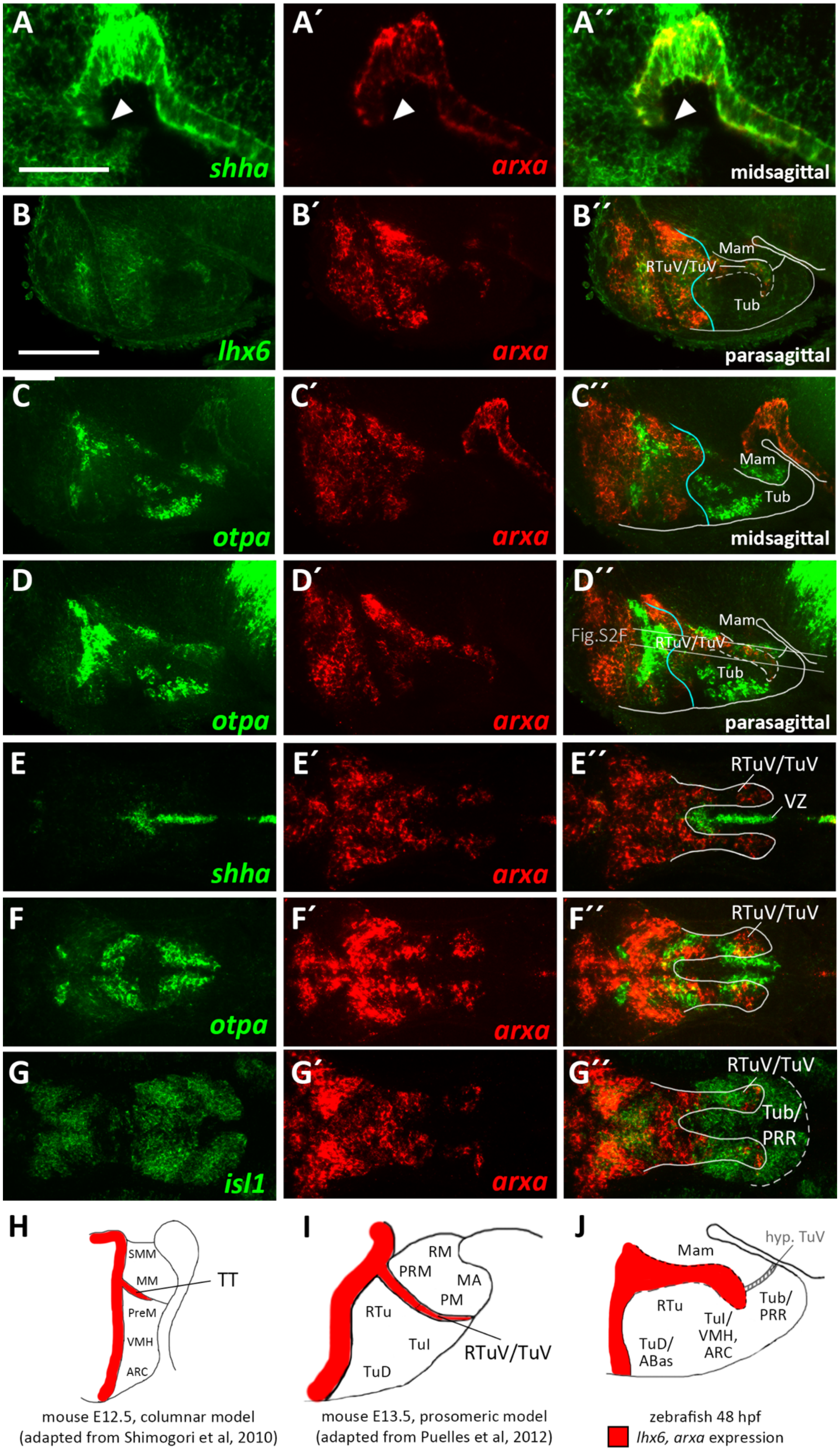
A
shha
A´
arxa
A´´
midsagittal
B
lhx6
B´
arxa
B´´
Mam
RTuV/TuV
Tub
parasagittal
C
otpa
C´
arxa
C´´
Mam
Tub
midsagittal
D
otpa
D´
arxa
D´´
Mam
Fig.S2F
RTuV/TuV
Tub
parasagittal
E
shha
E´
arxa
E´´
RTuV/TuV
VZ
F
otpa
F´
arxa
F´´
RTuV/TuV
G
isl1
G´
arxa
G´´
RTuV/TuV
Tub/
PRR
H
SMM
MM
TT
PreM
VMH
ARC
mouse E12.5, columnar model
(adapted from Shimogori et al, 2010)
I
RM
PRM
MA
PM
RTu
TuI
TuD
RTuV/TuV
mouse E13.5, prosomeric model
(adapted from Puelles et al, 2012)
J
hyp. TuV
Mam
RTu
Tub/
PRR
TuI/
VMH,
ARC
TuD/
ABas
zebrafish 48 hpf
lhx6, arxa expression

Figure S2. ***arxa*** **expression in floor plate and hypothalamus** (A-D´´) Sagittal and (E-G´´) horizontal optical sections of zebrafish embryos 48 hpf, stained by double-fluorescent whole-mount *in situ* hybridization using probes as indicated. (A-A´´) *arxa* and *shha* expression mark the floor plate (magnified views of Figure 1A-A´´). (B-J) The ventralmost tuberal domains can be identified by *arxa* or *lhx6* expression. Arrowhead in A´´ indicates the rostral border of the floor plate. The alar-basal boundary is shown in cyan. (H) *Arx* and *Lhx6* expression domain (red) within the E12.5 mouse hypothalamus, organized according to the columnar model after Shimogori et al., 2010. (I) Expression of *Arx* and *Lhx6* (red) within the E13.5 mouse basal hypothalamus organized according to the updated prosomeric model after Puelles et al., 2012. (J) Expression of *arxa* and *lhx6* within the basal hypothalamus of zebrafish embryos 48 hpf as observed in this study. Dashed gray area indicates the hypothetical course of the TuV domain (hyp. TuV) as predicted by the prosomeric model, which was, however, not detected through *lhx6* and *arxa* expression in our study (see main text for details). Maximum intensity projections of 10 (A-A´´D-F´´), 25 (B-B´´), 15 (C-C´´) or 40 (G-G´´) 1 µm confocal planes. Abbreviations see list in main text. Scale bar 50 µm (A), 100 µm (B; valid for B-G´´).

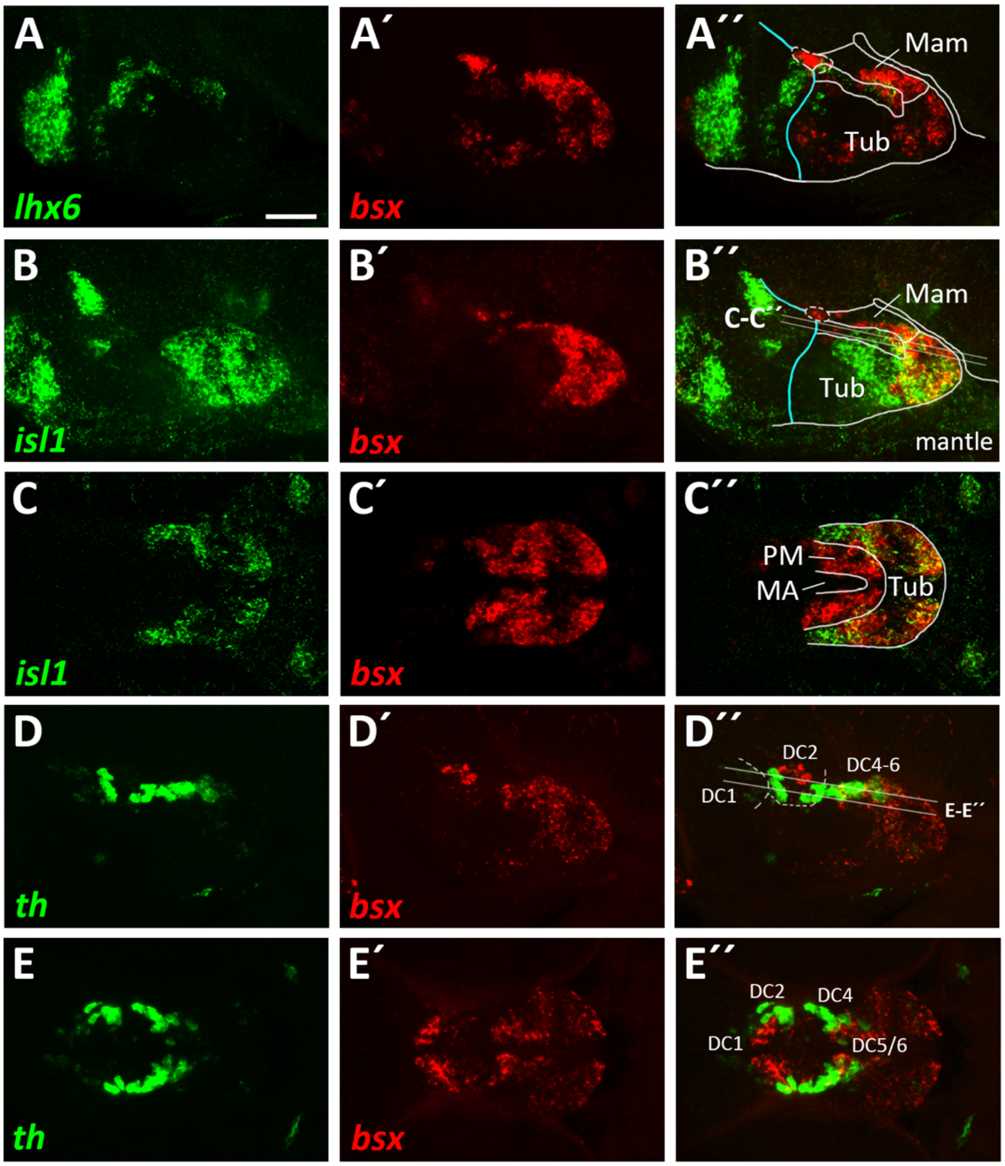

Figure S3: **Expression of *bsx* in relation to other genes in the tuberal and mamillary hypothalamus**. (A-B´´, D-D´´) Sagittal and (C-C´´, E-E´´) horizontal optical sections of zebrafish embryos 48 hpf, stained by double-fluorescent whole-mount *in situ* hybridization using probes as indicated. The alar-basal boundary is shown in cyan. Maximum intensity projections of 30 (A-A´´), 10 (B-C´´), 40 (D-E´´) 1 µm confocal planes. Abbreviations see list in main text. Scale bar 50 µm.

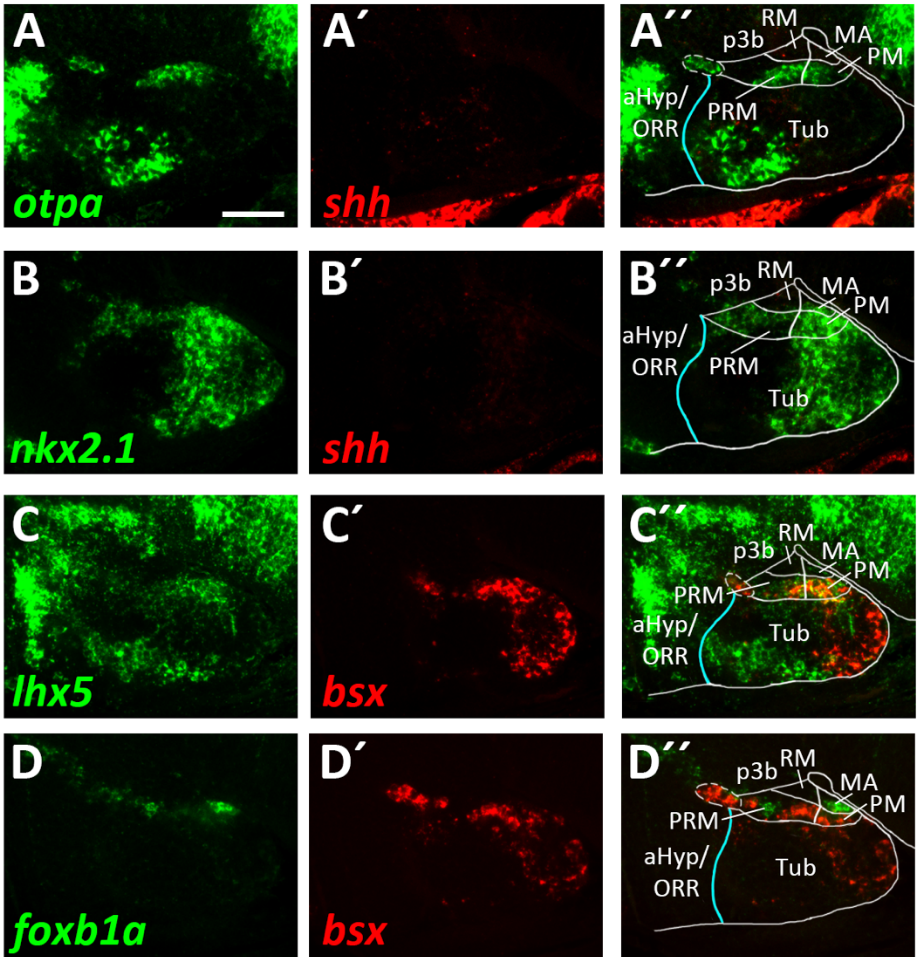

Figure S4. Borders of mamillary regions in parasagittal optical sections of the basal hypothalamus. (A-D´´) Sagittal optical sections of zebrafish embryos 48 hpf, stained by double-fluorescent whole-mount *in situ* hybridization using probes as indicated. All panels show maximum intensity projections of 10 adjacent confocal planes with 1 μm thickness. The alar-basal boundary is shown in cyan. More ventricular, i.e. median level projections are shown in Figure 3. Abbreviations see list in main text. Scale bar 50 μm.

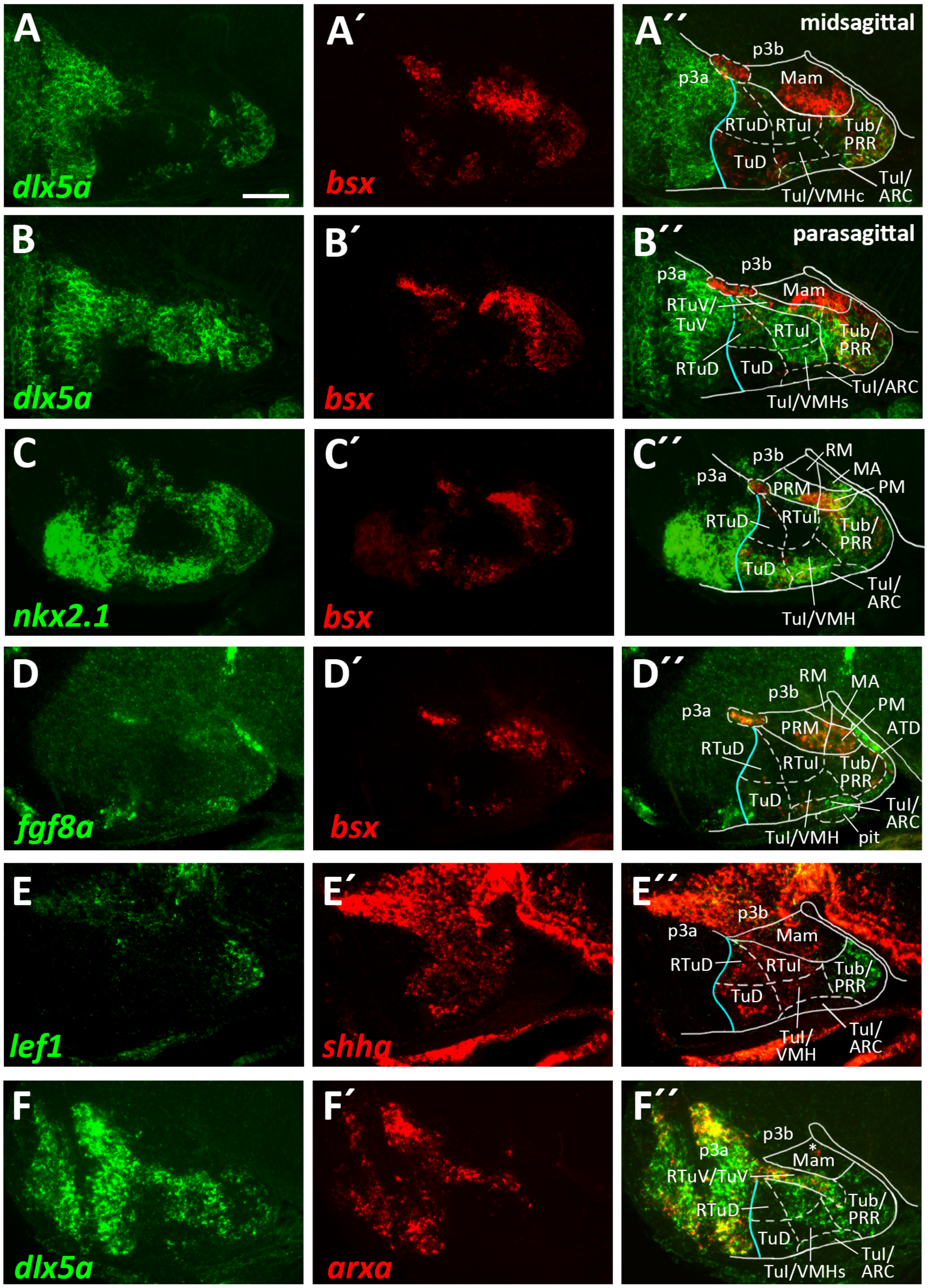

Figure S5. Subdivisions of tuberal hypothalamic regions as identified by gene expression domains. (A-F´´) Sagittal optical sections of zebrafish embryos 48 hpf, stained by double-fluorescent whole-mount *in situ* hybridization using probes as indicated. Maximum intensity projections of 30 (A-A´´, C-D´´), 15 (B-B´´, E-E´´) or 20 (F-F´´) 1 µm confocal planes. Asterisk in F´´ indicates *arxa* expression in the floor plate. The alar-basal boundary is shown in cyan. ATD – acroterminal domain; VMHc – ventromedial hypothalamus, core region; VMHs – ventromedial hypothalamus, shell region. For all other abbreviations see list in main text. Scale bar 50 µm.

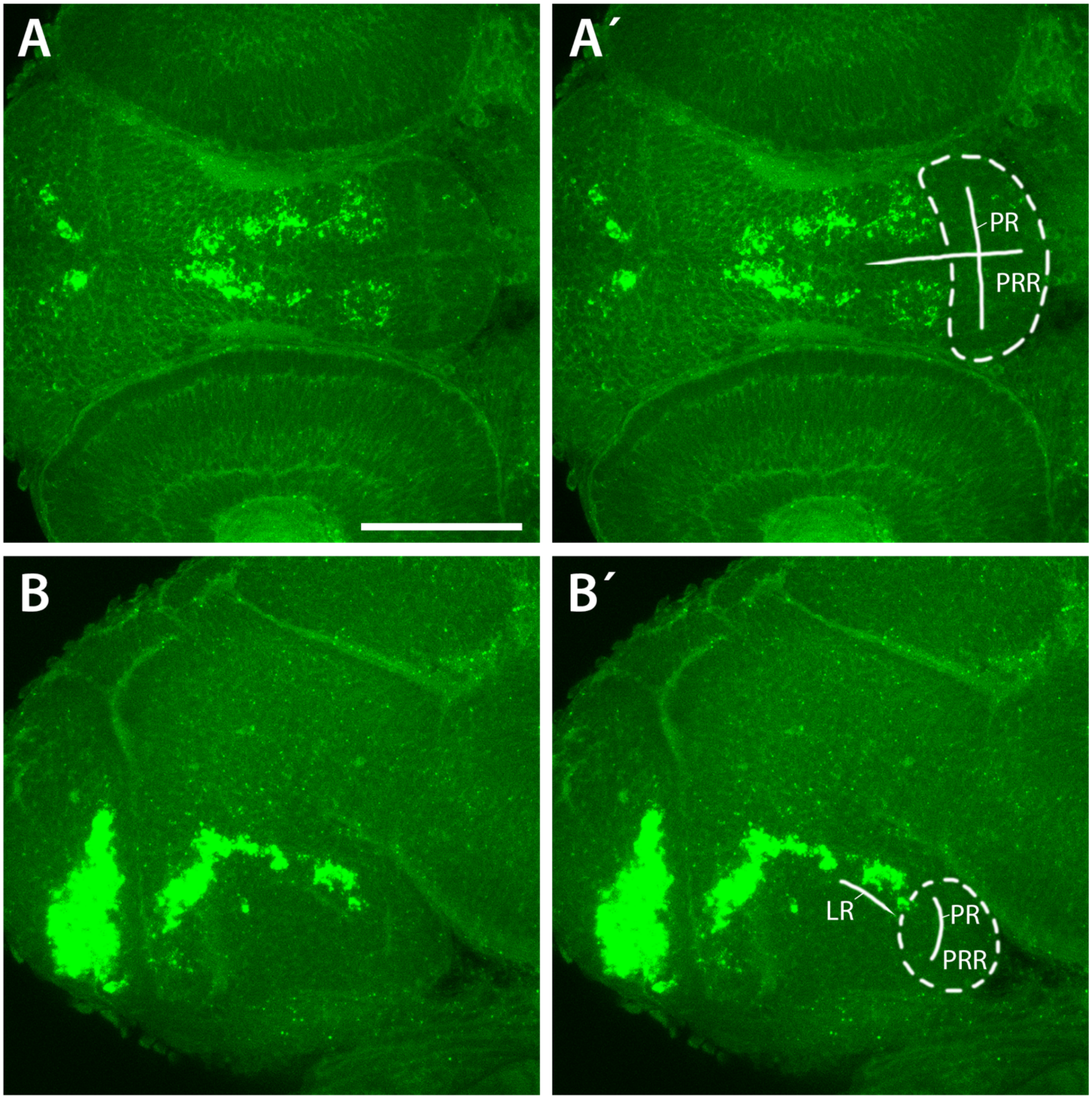

Figure S6. Expression of *lhx6* in relation to ventricular recesses. Horizontal and sagittal optical sections of zebrafish embryos 48 hpf stained by fluorescent whole-mount *in situ* hybridization using *lhx6* probe. Histograms were clipped in a way that the tissue contrast is high to allow detection of ventricular recesses. Maximum intensity projections of 13 (A-A′) or 22 (B-B′) 1 µm confocal planes. Anterior (in relation to body axis) is to the left. LR – lateral recess; PR – posterior recess; PRR – posterior recess region. Scale bar 100 µm.

Abbreviation	Reference / Primer Sequences	Enzyme	Polymerase
agrp	received from Matthias Hammerschmidt Lab, Cologne; verified by sequencing	BamHI	T7
arxa	(Miura et al., 1997)	SalI	SP6
bsx	(Schredelseker and Driever, 2018)	BamHI	T7
dlx5a	fwd: 5'-GTGCGTAACAGCGCAATTTAGG-3'<br>rev: 5'-TGCACTCTGTTATATGTTCCTGCG-3'	NotI	SP6
fgf8a	(Fürthauer et al., 1997)	NotI	T7
foxb1a	(Thisse et al., 2001)	EcoRI	T7
isl1	(Tokumoto et al., 1995)	XbaI	T3
lef1	fwd: 5'-AGCACGACACAGACCTGATG-3'<br>rev: 5'-GAAAAAGACGCCCGCTTCCTCC-3'	NotI	SP6
lhx5	(Peng and Westerfield, 2006)	SpeI	T7
lhx6	fwd: 5'-TGAAGTTACGCCGTCAAATG-3'<br>rev: 5'-GTCAAGAGCAGGACCTCTGG-3'	NotI	T3
lhx9	fwd: 5'-AAGGCGAAAGAAAGCAGTTGC-3'<br>rev: 5'-CCATTTCCGAAGCGGATATGC-3'	BamHI	T7
nkx2.1	fwd: 5'-GTGCCTGTGTTGGTGAAAGA-3'<br>rev: 5'-TCACCACGTCCTGCCATA-3'	SpeI	T7
nkx2.2a	fwd: 5'-ATTAACTGTGCGGTGTCTCAACG-3'<br>rev: 5'-CCGTTGCTTGAAGTTTCCTTGTC-3'	BamHI	T7
nr5a2	received from Matthias Hammerschmidt Lab, Cologne; verified by sequencing	KpnI	T3
otpa	fwd: 5'-AAGAAGACCACCAACGTGTTCCG-3'<br>rev: 5'-AGGTGAAGCTCATGGACACTGTG-3'	BamHI	T7
pax6a	(Krauss et al., 1991)	BamHI	T7
pax7a	(Seo et al., 1998)	BamHI	T7
penka	fwd: 5'-GGTGGACTGTGGCTTTGAG-3'<br>rev: 5'-TCCTTCCTCCCAAGTGCG-3'	NotI	SP6
shha	(Ekker et al., 1995)	HindIII	T7
th	(Holzschuh et al., 2001)	XhoI	T3

Table S1: All Genes for which RNA antisense probes were used for whole-mount *in situ* hybridization. In the second column, references are given for all probes that have been published previously and primer sequences are given for cDNA fragments that were cloned during this study. Enzymes used for linearization of template plasmids and RNA polymerase used for *in vitro* transcription of DIG-labelled RNA antisense probes are listed. Full names of genes and NCBI RefSeq including start and end position of probe sequences can be found in Table 1.

Supplementary References

Ekker, S. C., Ungar, A. R., Greenstein, P., Kessler, D. P. von, **Porter, J. A., Moon, R. T. and Beachy, P. A.** (1995). Patterning activities of vertebrate hedgehog proteins in the developing eye and brain. *Current Biology* **5**, 944–955. doi:10.1016/S0960-9822(95)00185-0.

Fürthauer, M., Thisse, C. and Thisse, B. (1997). A role for FGF-8 in the dorsoventral patterning of the zebrafish gastrula. *Development* **124**, 4253–4264.

Holzschuh, J., Ryu, S., Aberger, F. and Driever, W. (2001). Dopamine transporter expression distinguishes dopaminergic neurons from other catecholaminergic neurons in the developing zebrafish embryo. *Mechanisms of Development* **101**, 237–243. doi:10.1016/S0925-4773(01)00287-8.

Krauss, S., Johansen, T., Korzh, V. and Fjose, A. (1991). Expression of the zebrafish paired box gene pax[zf-b] during early neurogenesis. *Development* **113**, 1193.

Miura, H., Yanazawa, M., Kato, K. and Kitamura, K. (1997). Expression of a novel aristaless related homeobox gene 'Arx' in the vertebrate telencephalon, diencephalon and floor plate. *Mechanisms of Development* **65**, 99–109.

Peng, G. and Westerfield, M. (2006). Lhx5 promotes forebrain development and activates transcription of secreted Wnt antagonists. *Development* **133**, 3191–3200. doi:10.1242/dev.02485.

Puelles, L., Martinez-de-la-Torre, M., Bardet, S. and Rubenstein, J. L. R. (2012). Chapter 8 - Hypothalamus. In *The Mouse Nervous System* (ed. C. Watson, G. Paxinos and L. Puelles), pp. 221–312. San Diego: Academic Press.

Schredelseker, T. and Driever, W. (2018). Bsx controls pineal complex development. *Development* **145**. doi:10.1242/dev.163477.

Seo, H.-C., Sætre, B. O., Håvik, B., Ellingsen, S. and Fjose, A. (1998). The zebrafish Pax3 and Pax7 homologues are highly conserved, encode multiple isoforms and show dynamic segment-like expression in the developing brain. *Mechanisms of Development* **70**, 49–63. doi:10.1016/S0925-4773(97)00175-5.

Shimogori, T., Lee, D. A., Miranda-Angulo, A., Yang, Y., Wang, H., Jiang, L., Yoshida, A. C., Kataoka, A., Mashiko, H. and Avetisyan, M. et al. (2010). A genomic atlas of mouse hypothalamic development. *Nature neuroscience* **13**, 767–775. doi:10.1038/nn.2545.

Thisse, B., Pflumio, S., Fürthauer, M., Loppin, B., Heyer, V., Degrave, A., Woehl, R., Lux, A., Steffan, T. and Charbonnier, X. Q. et al. (2001). Expression of the zebrafish genome during embryogenesis. ZFIN Direct Data Submission. https://zfin.org/ZDB-PUB-010810-1.

Tokumoto, M., Gong, Z., Tsubokawa, T., Hew, C. L., Uyemura, K., Hotta, Y. and Okamoto, H. (1995). Molecular Heterogeneity among Primary Motoneurons and within Myotomes Revealed by the Differential mRNA Expression of Novel Islet-1 Homologs in Embryonic Zebrafish. *Developmental Biology* **171**, 578–589. doi:10.1006/dbio.1995.1306.
